# Functional Gene Composition, Diversity and Redundancy in Microbial Stream Biofilm Communities

**DOI:** 10.1371/journal.pone.0123179

**Published:** 2015-04-07

**Authors:** Andrew Dopheide, Gavin Lear, Zhili He, Jizhong Zhou, Gillian D. Lewis

**Affiliations:** 1 School of Biological Sciences, University of Auckland, Auckland, New Zealand; 2 Institute for Environmental Genomics and Department of Microbiology and Plant Biology, University of Oklahoma, Norman, OK, United States of America; Missouri University of Science and Technology, UNITED STATES

## Abstract

We surveyed the functional gene composition and diversity of microbial biofilm communities in 18 New Zealand streams affected by different types of catchment land use, using a comprehensive functional gene array, GeoChip 3.0. A total of 5,371 nutrient cycling and energy metabolism genes within 65 gene families were detected among all samples (342 to 2,666 genes per stream). Carbon cycling genes were most common, followed by nitrogen cycling genes, with smaller proportions of sulphur, phosphorus cycling and energy metabolism genes. Samples from urban and native forest streams had the most similar functional gene composition, while samples from exotic forest and rural streams exhibited the most variation. There were significant differences between nitrogen and sulphur cycling genes detected in native forest and urban samples compared to exotic forest and rural samples, attributed to contrasting proportions of nitrogen fixation, denitrification, and sulphur reduction genes. Most genes were detected only in one or a few samples, with only a small minority occurring in all samples. Nonetheless, 42 of 65 gene families occurred in every sample and overall proportions of gene families were similar among samples from contrasting streams. This suggests the existence of functional gene redundancy among different stream biofilm communities despite contrasting taxonomic composition.

## Introduction

The role of streams as landscape drainage systems makes these ecosystems susceptible to impacts of land use changes such as deforestation, agriculture, and urbanisation [1–3]. While there is much interest in improving the health of degraded streams, our understanding of stream ecological processes is incomplete, limiting the effectiveness of management and restoration efforts. In particular, the links between microbial community composition, metabolic functions, and stream biogeochemistry are not well understood [4].

A majority of the diverse and abundant microorganisms found in streams occur in surface-associated biofilm communities, where they are thought to contribute significantly to in-stream ecological processes [5, 6]. This includes carbon (C) processing [7–9], nitrogen (N) and sulphur (S) cycling [10], and immobilization and transformation of aquatic contaminant molecules [11–13]. Catchment land use affects the organic material present in streams [14, 15], and streams in urban and agricultural catchments typically receive elevated inputs of nutrients and inorganic pollutants [1, 16]. Recent studies have revealed differences in the composition of stream benthic bacterial communities related to catchment urbanization [17, 18], and evidence suggests such changes in microbial community composition may influence microbially-mediated functions [8, 18–22]. However, it remains unclear to what extent differences in microbial community composition in stream biofilms are accompanied by differences in microbial functional genes or associated in-stream biogeochemical processes.

The study of microbial functional genes in natural environments is challenging due to the great diversity and complexity of microbial communities and associated functional genes. However, recently developed molecular approaches are providing new insights into the role of microbial functional genes in natural systems [23]. For example, GeoChip 3.0 is a comprehensive functional gene microarray which includes 27,812 probes covering 56,990 gene sequences from 292 functional gene families involved in C, N, S and phosphorus (P) cycling, energy metabolism, antibiotic and metal resistance, organic contaminant degradation, and a phylogenetic marker gene [24].

In the present study, we surveyed the microbial functional gene composition of biofilm samples from a variety of streams affected by different land use types using GeoChip 3.0. Our objectives were to characterize the typical functional gene composition of microbial stream biofilm communities, to investigate whether there were any differences in functional gene composition among streams representing different types of catchment land use, and to explore links between microbial community structure and environmental variables. The present study focuses on the functional genes involved in nutrient (C, N, P and S) cycling and energy metabolism. We detected a broad range of microbial nutrient cycling and energy metabolism genes in stream biofilms, evidence of contrasting composition among streams affected by different types of catchment land use (depending on functional gene category), and the apparent existence of functional gene redundancy despite contrasting microbial composition.

## Materials and Methods

### Sample sites and sample collection

Biofilm samples were collected in November 2009 from 18 streams and rivers located throughout the greater Auckland region in New Zealand (Table 1). These streams are located in catchments representing the dominant types of land development in the region, with varying proportions of forest, agriculture, and urban land cover, and with in-stream water quality and ecological health ranging from excellent to poor, according to results of a long-term monitoring programme [25–27]. Prior investigations have found evidence of catchment development and water quality-related differences in microbial biofilm communities among these streams [28]. The locations of sampling sites were kindly provided by the Freshwater Monitoring group at Auckland Council. The sites and sampled material are not protected, and no special permits were required for sampling at any of the sites.

**Table 1:** Catchment and site attributes of the streams sampled for this study.

Stream	Site Location		Catchment area (ha)^1^	Land use category^1^	Catchment land use (%)^1^						Shade^1^	Water quality^2^
	Latitude	Longitude			Native	Exotic	Pasture	Hort.	Urban	Other		
Cascade Stream (CASC)	36 53 19.8 S	174 31 19.4 E	1390	Native forest	98.4	-	-	-	-	1.6	-	Excellent
Opanuku Stream (OPAN)	36 53 42.2 S	174 35 40.8 E	1568	Native forest	82.2	0.2	15.6	1.0	1.0	-	4.5 / 3.3	Excellent
Mahurangi River (MAHU)	36 27 00.4 S	174 38 50.3 E	486	Exotic forest	1.5	98.2	0.3	-	-	-	5.0 / 3.8	Good
Riverhead Stream (RIVE)	36 44 09.5 S	174 32 08.7 E	413	Exotic forest	0.3	96.8	0.8	-	-	2.0	7.0 / 5.8	Good
Hoteo River (HOTE)	36 22 57.9 S	174 30 29.8 E	26728	Rural	19.4	23.3	56.6	0.2	0.3	0.2	-	Good
Kumeu River (KUME)	36 46 35.5 S	174 33 37.6 E	4568	Rural	12.3	4.0	77.0	4.7	1.9	0.1	1.5 / 2.3	Good
Makarau River (MAKA)	36 33 27.4 S	174 31 16.9 E	2137	Rural	20.6	3.5	75.5	-	-	0.5	2.5 / 4.7	Good
Matakana River (MATA)	36 20 40.2 S	174 42 37.9 E	1340	Rural	41.0	19.0	39.7	0.1	0.2	-	6.5 / 6.8	Good
Ngakaroa Stream (NGAK)	37 11 43.5 S	174 58 25.0 E	448	Rural	1.4	3.7	74.2	20.7	-	-	3.5 / 3.7	Fair
Okura Creek (OKUR)	36 41 06.5 S	174 41 40.6 E	552	Rural	54.3	9.7	35.8	-	0.1	-	-	Poor
Rangitopuni River (RANG)	36 44 38.7 S	174 37 04.4 E	8369	Rural	10.9	16.6	69.2	2.2	0.5	0.6	-	Good
Lucas Creek (LUCA)	36 43 22.4 S	174 41 57.9 E	609	Urban	8.6	8.7	40.4	-	40.9	1.3	6.5 / 2.0	Good
Oakley Creek (OAKL)	36 52 30.0 S	174 42 18.1 E	1224	Urban	2.0	0.1	-	-	97.9	-	3.5 / 4.6	Fair
Omaru Creek (OMAR)	36 52 49.0 S	174 51 37.9 E	472	Urban	0.7	-	-	-	77.3	22.0	1.5 / 2.9	Fair
Otara Creek (OTAR)	36 57 20.4 S	174 53 26.6 E	1218	Urban	5.7	1.7	83.8	0.4	8.4	0.1	6.5 / 7.6	Fair
Oteha Stream (OTEH)	36 44 13.2 S	174 42 11.4 E	1831	Urban	6.9	3.4	13.6	-	69.7	6.4	9.0 / 8.2	Good
Pakuranga Creek (PAKU)	36 54 46.5 S	174 54 54.3 E	793	Urban	0.4	0.3	0.2	-	99.1	-	0.0 / 0.0	Poor
Puhinui Stream (PUHI)	36 59 34.0 S	174 52 13.2 E	1043	Urban	9.9	1.6	45.2	-	43.1	0.1	1.0 / 1.0	Poor

^1^ Catchment and shade data are provided by Auckland Council (http://www.aucklandcouncil.govt.nz). Catchments which are predominantly forested with very little or no urban development are classified as either native forest or exotic forest according to forest composition. Catchments with little or no urban development and a mix of forest, pastoral, or horticultural land use are classified as rural. Catchments with ≥ 10% urban development (approximate) are classified as urban. Each pair of shade values represents the amount of shading upstream of the sampling site scored out of ten, and the extent of shading at the sampling site scored out of ten (0 = no shading, 10 = complete shading).

^2^ Water quality descriptions represent the results of monthly monitoring of a suite of chemical water quality parameters throughout 2009 [25].

At each sampling site, the stream was divided into five consecutive reaches of approximately 5 m length. Five rocks were randomly selected and consecutively removed from within each reach, starting from the most downstream reach and proceeding in an upstream direction, for a total of 25 rocks per stream. After removal from the water, biofilm was immediately sampled from the upper surface of each rock by abrasion with a sterile Speci-sponge (Nasco, Fort Atkinson, WI, USA), which was then sealed in a sterile Whirl-Pak bag (Nasco) and chilled on ice, and the rock returned to the stream. In one stream (Oteha stream) no rocks were found, and in this case samples were instead collected from fallen tree branches.

In the laboratory, each Speci-sponge was transferred into a bag with about 40 ml sterile water, and subjected to repetitive compression in a Seward Stomacher laboratory blender (normal speed, 120 s) to release biofilm material. The released material was transferred into 50 mL centrifuge tubes and pelleted by centrifugation (3500 x g, 10 min). Pelleted biofilm samples from the five rocks (or branches, for Oteha Stream) within each stream reach were combined, resulting in five samples per stream, and stored at -20°C until DNA extraction.

### DNA preparation and GeoChip hybridization

DNA was extracted from each of the five combined biofilm samples from each stream (0.25 g) using the method of Zhou et al. [29] and resuspended in 100 μl ultra pure water (Invitrogen). DNA extracts from the five biofilm samples from each stream were pooled to give a single representative DNA sample for each stream. Pooled DNA extracts were then subjected to whole-genome amplification using a Templiphi Kit (GE Healthcare, Piscataway, NJ, USA) and a modified buffer which included single-strand binding protein (267 ng μl^-1^) and spermidine (0.1 mM) to improve amplification efficiency [30]. Reactions were incubated at 30°C for three hours then stopped by heating to 65°C for 10 minutes. Amplification products were subsequently labeled with the fluorescent dye Cy-5 using a random priming method, as described previously [31], before purification using a QIA quick purification kit (Qiagen, Valencia, CA, USA) and drying in a SpeedVac (Thermosavant, Milford, MA, USA) at 45°C for 45 minutes. Samples were then resuspended in 120 μl hybridization buffer (50% formamide, 3 x saline-sodium citrate buffer, 10 μg herring sperm DNA (Promega, Madison, WI, USA) and 0.1% SDS), denatured at 95°C for 5 minutes, and then hybridized with a GeoChip 3.0 microarray on an HS4800 Pro hybridization station (TECAN US, Durham, NC, USA) according to manufacturer’s directions. The hybridized microarrays were scanned using a Scan Array Express Microarray Scanner (Perkin-Elmer, Boston, MA, USA), and resulting images processed using ImaGene 6.0 software (BioDiscovery, El Segundo, CA, USA) according to previously described procedures [32]. To assess the reproducibility of this microarray-based approach, biofilm samples from six streams (Cascade, Opanuku, Hoteo, Matakana, Lucas and Oakley) were subjected to triplicate GeoChip hybridizations, while all other samples were hybridized once.

### Data analysis

GeoChip data comprised a list of functional genes detected in each stream, with the abundance of each gene indicated by the measured intensity of the probe hybridization signal. Shannon and Simpson diversity indices were initially calculated on raw gene abundance data. For comparisons of overall functional gene assemblages, total gene abundance data was standardized among samples. Alternatively, for comparisons of the relative proportions of functional gene families, the numbers of detected genes belonging to different gene families were summed and expressed as proportions of the total number of genes in each sample. Subsets of data belonging to different functional gene categories (carbon, nitrogen, phosphorus, and sulphur cycling, and energy metabolism) were standardized and analysed separately. For analysis of the taxonomic composition of samples, the number of *gyrB* phylogenetic marker genes belonging to different genera were summed and expressed as proportions of the total number of *gyrB* genes detected in each sample. The *gyrB* gene encodes the DNA gyrase β-subunit, and provides a higher level of taxonomic resolution than 16S ribosomal RNA gene sequences [24, 33, 34].

Similarities and differences between standardized and proportional gene assemblage data from different streams and catchment types were investigated using multivariate statistical analyses. Bray-Curtis similarity of gene assemblages was calculated between all samples. Differences between samples were investigated using clustering analysis, non-metric multidimensional scaling (MDS) and Analysis of Similarity (ANOSIM). Similarity Percentage (SIMPER) analysis was used to investigate the variables contributing to observed similarities and differences. Similarities between patterns of Bray-Curtis sample similarity based on different subsets of functional genes and based on taxonomic composition were investigated using RELATE (a Mantel-type comparison of similarity matrices). All multivariate statistical analyses (MDS, ANOSIM, SIMPER, and RELATE) were carried out in Primer 6 (Primer-E Ltd., UK). Links between functional gene assemblages and environmental variables were investigated by regressing water quality and catchment land use measurements against functional gene diversity indices, and against primary and secondary axes of MDS plots based on functional gene assemblages in biofilm samples. Regression analyses were carried out in R 2.12.1 [35].

## Results

### Reproducibility of GeoChip analysis of stream biofilm samples

To investigate the reproducibility of the GeoChip analysis method, pooled biofilm DNA samples from six streams were each subjected to triplicate GeoChip hybridizations. Triplicate results from three streams (Cascade, Opanuku, and Lucas) had very similar numbers and composition of genes (≥ 90% of detected genes shared between triplicate results; > 80% Bray-Curtis similarity between triplicate results). Triplicate results for each of the remaining three streams (Matakana, Hoteo, and Oakley) had similarly consistent gene assemblages, except one triplicate result in each case had 20% to 40% fewer genes than the other two triplicate results, suggesting reduced GeoChip hybridization effectiveness in these cases. Clustering analysis showed that triplicate hybridization results from each stream always grouped together however, with the exception of one result from Oakley Creek biofilm (S1 Fig). ANOSIM analysis indicated that triplicate analysis results from each stream were always significantly more similar to each other than they were to results from different streams (Global R = 0.863, p = 0.001; all pairwise R values ≥ 0.889, p = 0.1). This suggests that the results of GeoChip analysis of stream biofilms are generally repeatable, providing confidence in the results of non-triplicate analyses.

Fewer than 400 genes were detected by triplicate analyses of Matakana Stream biofilm and by one of three analyses of Hoteo River biofilm. This represents less than half of the number of genes detected in any other samples, and these samples were excluded from further analysis. All other triplicate results were subsequently replaced with single results by averaging the probe hybridization data among triplicate results, or by calculating distances among centroids in Primer 6 (Primer-E Ltd., UK) for subsequent multivariate analyses.

### Genes and gene families detected in stream biofilms

In total, 12,801 genes and 273 gene families were detected among all biofilm samples, corresponding to 46% of the probes and 93.5% of the gene families represented on the GeoChip 3.0 microarray (S4 Table). Of these, 5,371 genes (65 gene families) were associated with nutrient (C, N, P, and S) cycling and energy metabolism. A further 638 genes were *gyrB* phylogenetic markers for analysis of taxonomic composition. The remaining 6,792 genes (206 gene families) were associated with resistance to antibiotics and metals or remediation of organic contaminants. This paper focuses on the nutrient cycling, energy metabolism, and phylogenetic marker genes.

The number of nutrition and energy metabolism genes detected in biofilm samples varied widely, from 342 from rural Ngakoroa Stream to 2,666 from rural Kumeu Stream, with a mean of 940 genes detected per stream (sd = 570; Table 2). There was no evidence of differences in the numbers of genes detected in samples from streams with different predominant types of catchment land use. About half of the 5,371 nutrition and energy metabolism genes were each detected only in one sample, about half again were detected in two samples, and there were similar reductions between the numbers of genes shared between three, four, five or six samples. Between 46 and 81 genes were shared between each of seven to 17 samples. Genes that were detected only once accounted for 1.4% to 13% of the genes detected in most samples, with the remainder detected in two or more samples. Biofilm samples from two streams (rural Kumeu Stream and urban Oakley Creek) had unusually high proportions of unique genes however (35% and 58% respectively), which together accounted for one third of the total number of genes detected among all samples. There were 31 genes detected only in samples from native forest streams, compared with 190 genes only detected in exotic forest streams, 1,562 genes detected only in rural streams, and 1,222 genes detected only in urban streams. On average, any two samples shared 48% of their genes (sd = 19.2%).

**Table 2:** Number, diversity and composition of nutrition and energy metabolism genes, *gyrB* genes, and number of gene families detected in stream biofilm samples.

		Stream																	Geochip
		CASC	OPAN	MAHU	RIVE	HOTE	KUME	MAKA	NGAK	OKUR	RANG	LUCA	OAKL	OMAR	OTAR	OTEH	PAKU	PUHI	
Nutrition/energy genes	Number of genes	812	508	974	898	446	2666	688	342	1735	833	853	1551	808	832	765	460	813	22345
	Unique genes (%)	1.4	3.9	7.4	12.2	13.7	34.8	10.0	5.0	13.4	5.5	2.2	57.8	5.7	8.3	5.5	5.4	8.5	-
	Shannon diversity	6.38	5.94	6.49	6.53	5.98	7.34	6.08	5.45	7.09	6.40	6.43	7.45	6.32	6.37	6.29	5.80	6.32	-
	Simpson’s Reciprocal index	430	238	418	468	299	1108	275	146	785	429	427	1226	366	401	357	223	371	-
	Carbon cycling (%)	46.9	46.2	47.3	43.3	63.9	45.0	48.0	44.2	45.7	47.3	46.4	65.4	47.4	43.9	43.5	47.2	44.0	47.3
	Nitrogen cycling (%)	32.5	29.0	29.7	31.7	16.9	33.4	28.2	32.2	31.9	30.4	31.9	22.8	32.2	32.9	31.8	30.0	31.9	35.1
	Phosphorus cycling (%)	4.7	4.8	5.4	4.3	3.8	4.9	5.1	4.4	4.7	4.4	4.7	3.4	5.1	5.2	5.8	4.8	5.4	5.5
	Sulphur cycling (%)	12.4	14.8	12.0	14.8	6.2	12.8	13.7	14.6	12.7	12.6	12.7	4.3	11.5	12.9	14.8	14.8	13.0	9.1
	Energy metabolism (%)	3.5	5.2	5.5	5.8	9.2	4.0	5.1	4.7	5.0	5.3	4.3	4.1	3.8	5.2	4.2	3.3	5.7	3.0
	Number of gene families	59	56	58	57	52	65	59	49	62	59	60	60	57	56	58	53	60	66
*gyrB* genes	Number of genes	74	40	78	59	44	269	73	32	114	62	72	320	86	62	64	46	63	2298
	Unique genes (%)	0.0	0.0	10.3	10.2	16.1	29.7	21.9	9.4	7.9	3.2	0.0	77.2	1.2	25.8	1.6	2.2	6.3	-
	Shannon diversity^1^	3.96	3.51	4.04	3.81	3.67	5.03	3.82	3.14	4.28	3.79	3.97	5.97	4.16	3.81	3.82	3.53	3.80	-
	Simpson’s Reciprocal index	39.2	26.5	40.8	30.7	33.9	110.2	31.1	16.5	46.2	33.2	38.8	335.7	49.7	32.7	33.2	24.9	31.4	-

Unique genes represent the proportion of genes that were only detected in a single sample. The number of probes in each category on the Geochip 3.0 array is included for comparison.

The number of nutrient cycling (C, N, P and S) and energy metabolism gene families detected showed much less variation than the number of genes detected, ranging from 49 in rural Ngakaroa Stream biofilm to 65 gene families in rural Kumeu Stream biofilm (Table 2). A majority of the detected gene families (42 of 65) were present in all samples. Land use did not appear to select for any particular gene family.

The relative proportions of genes belonging to different overall nutrient cycling/energy metabolism categories were very similar between streams, with the exception of samples from the largest, rural waterway in the study, Hoteo River, and a stream with an almost entirely urban catchment, Oakley Creek (Table 2). C cycling genes constituted ≥ 64% of the nutrition/energy metabolism genes detected in these two samples, but 43%- 48% of the genes detected in other samples. Conversely, the proportion of genes associated with N cycling was 17% in Hoteo River biofilm and 23% in Oakley Creek, compared to 28%- 33% in all other samples. Both of these waterways also had less than half of the proportion of S cycling genes detected in most other samples, while Hoteo River biofilm also had the highest proportion of energy metabolism genes and Oakley Creek biofilm had a very high proportion of *gyrB* phylogenetic marker genes, most of which were not detected in any other samples. These proportional differences were consistent with differences of absolute gene abundance when samples with similar total gene abundance were compared.

### Functional gene composition differences between streams and catchment types

**Fig 1:**
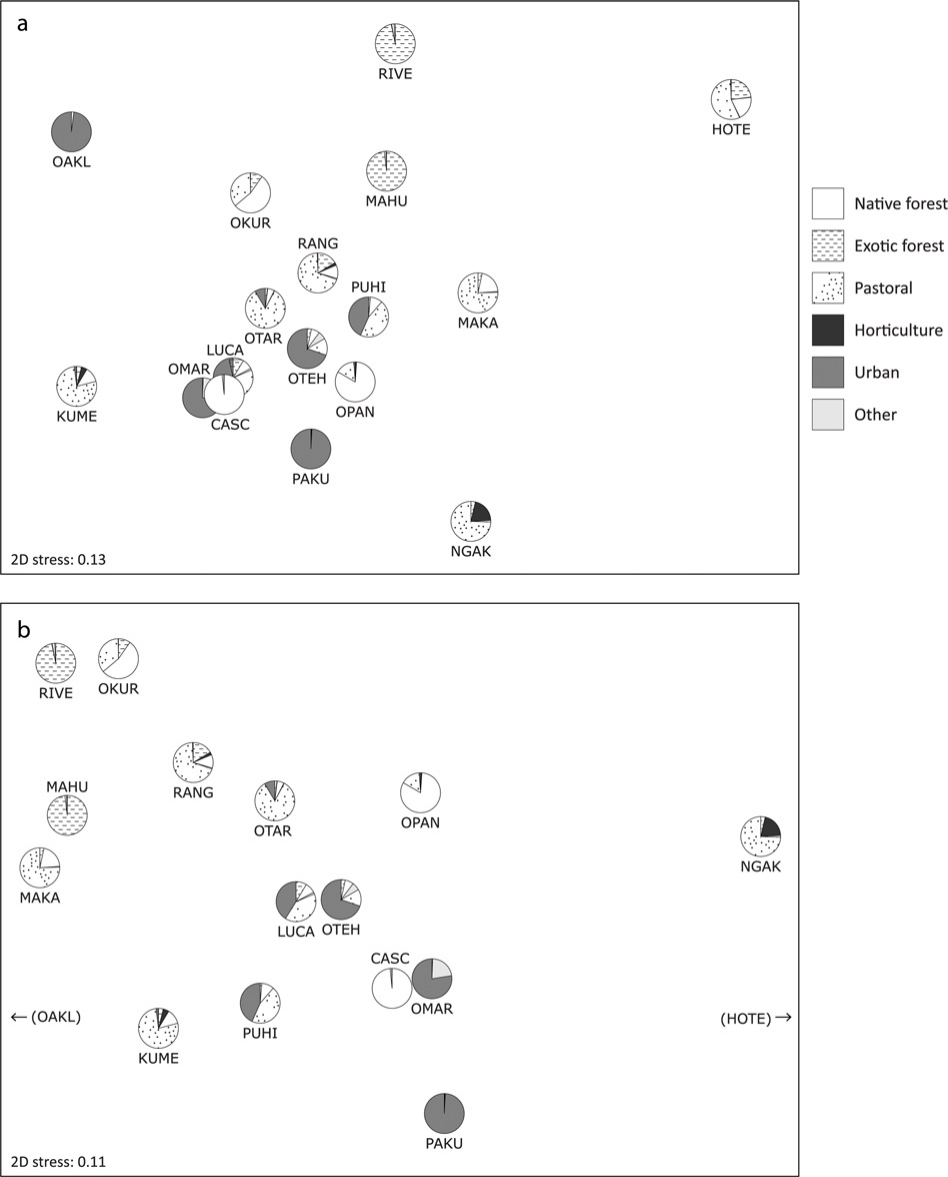
MDS plots based on microbial nutrient cycling and energy metabolism gene assemblages (a) and gene families (b) detected in stream biofilms by Geochip analysis. The total number of nutrition/energy metabolism genes detected in each sample was standardized between all samples (a), or the number of genes contributing to each of 65 nutrition/energy metabolism gene families was determined for each sample, and the total number of genes standardised between samples (b). Hoteo River and Oakley Creek samples had markedly distinct gene families to other samples resulting in a degenerate MDS plot when included, and have therefore been excluded from (b).

The relative Bray-Curtis similarity of biofilm samples based on the 5,371 nutrition/energy metabolism genes ranged from less than 20% to almost 80%, with a mean of 41.7% (sd = 12.3%). The greatest similarity (75–80%) was observed between biofilm from two urban streams (Lucas and Omaru) and, surprisingly, between these two streams and a native forest-dominated stream (Cascade), as indicated by their close proximity on an MDS plot (Fig 1a). In comparison, there was 58.1% similarity between gene assemblages detected in biofilm from two native forest streams with high water quality (Cascade and Opanuku). The lowest similarity was generally observed between rural Hoteo River biofilm compared to other streams (16.1%- 27.6% similarity), and between biofilm from highly urbanized Oakley Creek compared to other streams (23.3%- 38.6% similarity), as shown by the position of these sites on the periphery of the MDS ordination (Fig 1a). There was only 30% similarity between biofilm from two streams with near-wholly urban catchments (Oakley and Pakuranga). Low levels of gene assemblage similarity were also observed between biofilm from rural streams (Ngakaroa and Kumeu), and between biofilm from an exotic forest stream (Riverhead) compared to many other sites. Samples from native forest streams tended to group with those from most urban streams according to MDS ordination, suggesting relatively similar functional gene assemblages in these streams despite contrasting environmental characteristics (Fig 1a). Samples from rural streams were most scattered, and samples from exotic forest streams were separated from most other samples, suggesting divergent functional gene assemblages in these streams.

When biofilm samples were compared on the basis of relative proportions of 65 nutrition and energy metabolism gene families, Bray-Curtis similarity between most samples ranged from about 80% to over 90%, with the highest similarity again between Lucas, Omaru and Cascade samples, but markedly lower similarity between Hoteo and Oakley biofilm compared to other samples (62.1%- 68.7%). Exclusion of these two samples as outliers resulted in a relatively clear separation of native forest and urban stream samples from exotic forest and rural stream samples (Fig 1b). ANOSIM analysis suggested there were significant differences between functional gene composition of samples from exotic forest and urban streams, between rural and urban streams, and between native forest and exotic forest streams (Table 3). There was no evidence of significant differences between native forest and urban stream samples, or between rural and exotic stream samples.

**Table 3:** ANOSIM analysis results for comparisons of relative proportions of nutrition and energy metabolism gene families detected in stream biofilms.

	All nutrition and energy metabolism gene families^1^		Carbon cycling gene families^1^		Nitrogen cycling gene families		Sulphur cycling gene families	
	R statistic	P	R statistic	P	R statistic	P	R statistic	P
Global comparison	0.274	0.024*	0.032	0.398	0.267	0.018*	0.260	0.023*
Native—Exotic	1.0	0.333^†^	0.25	0.667	1.0	0.333^†^	1.0	0.333^†^
Native—Rural	0.145	0.286	-0.273	0.81	0.219	0.286	0.240	0.071
Native—Urban	-0.073	0.607	-0.104	0.643	0.032	0.472	-0.377	0.972
Exotic—Rural	-0.255	0.905	-0.382	1.0	-0.229	0.750	-0.125	0.786
Exotic—Urban	0.823	0.036*	0.156	0.357	0.727	0.028*	0.675	0.028*
Rural—Urban	0.323	0.004*	0.205	0.03	0.286	0.020*	0.354	0.006*

^1^Data from rural Hoteo River and urban Oakley Creek are excluded as outliers from all gene family comparisons and carbon gene family comparisons.

*Statistically significant p-values (p ≤ 0.05) are indicated.

^†^Native forest—exotic forest differences are considered probably significant due to significant global comparison differences, maximal pairwise R statistics, and pairwise p-values which are the lowest possible for the limited number of samples included.

The primary (horizontal) axis of the MDS ordination shown in Fig 1b showed a strong positive correlation (0.77, p = 0.001) with total in-stream N (S2 Table). The secondary (vertical) axis of Fig 1b was strongly negatively correlated with water temperature (-0.68, p = 0.006), and showed near-significant negative correlations with dissolved oxygen (-0.51, p = 0.054) and pH (-0.49, p = 0.062). In terms of land use, the primary axis of Fig 1b was positively correlated with the proportion of horticultural land use (0.64, p = 0.01) and negatively correlated with the proportion of exotic forest land use (-0.51, p = 0.051). The secondary axis of Fig 1b showed a strong positive correlation with shade (0.66, p = 0.02), and a strong negative correlation with urban land use (-0.63, p = 0.011). Just one significant correlation was detected between diversity indices and environmental parameters, with turbidity positively correlated with Shannon diversity of both nutrient cycling/energy genes (0.68, p = 0.003) and *gyr*B genes (0.59, p = 0.013).

### Carbon cycling genes

**Fig 2:**
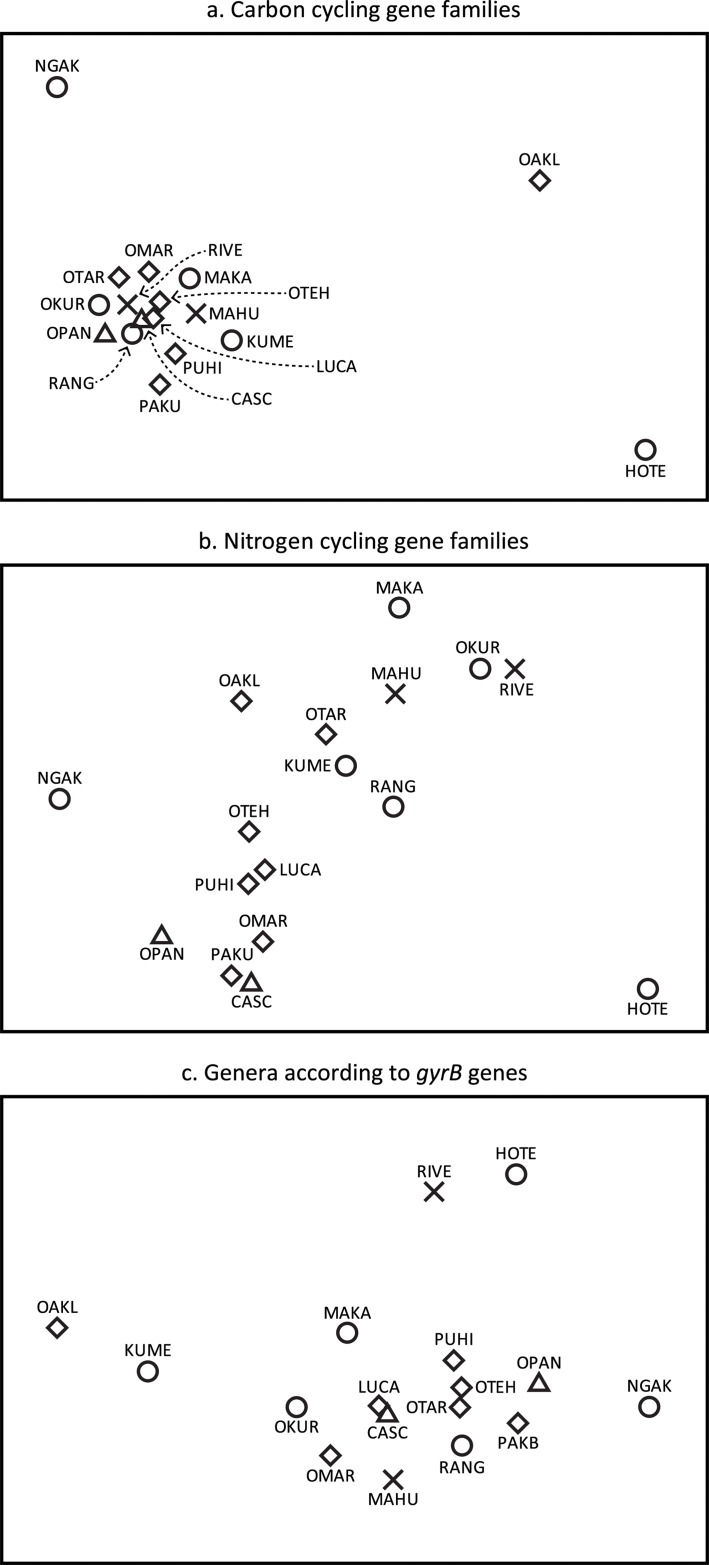
MDS plots based on the relative proportions of different C cycling gene families (a), N cycling gene families (b), and genera based on *gyrB* genes (c) detected by Geochip analysis of biofilm from native forest streams (△), exotic forest streams (⨯), rural streams (○) and urban streams (◇). The numbers of genes occurring within each C cycling (a) or N cycling (b) gene family were expressed as a proportion of the total number of C cycling or N cycling genes detected in each sample. The numbers of *gyrB* genes belonging to each detected genus were expressed as a proportion of the total number of *gyrB* genes detected in each sample (c).

**Fig 3:**
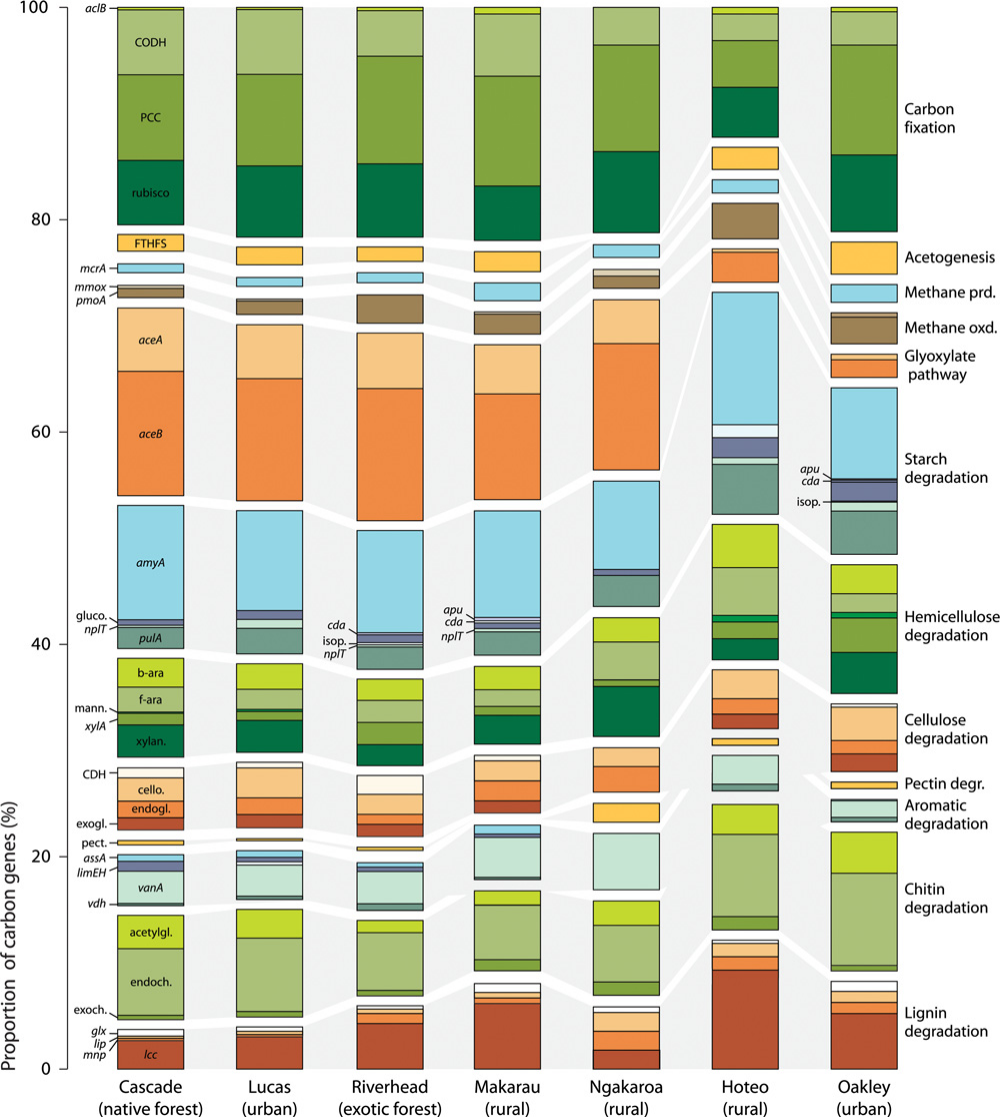
Relative proportions of C cycling gene families and categories detected in stream biofilm samples. Three samples with the most divergent C cycling gene assemblages (Ngakaroa, Hoteo, and Oakley streams) are shown in comparison to typical native forest, urban, exotic forest and rural streams. *aclB*, ATP citrate lyase; CODH, carbon monoxide dehydrogenase; PCC, propionyl-CoA/acetyl-CoA carboxylase; rubisco, ribulose-1, 5-bisphosphate carboxylase/oxygenase; FTHFS, formyltetrahydrofolate synthetase; *mcrA*, methyl coenzyme M reductase; *pmoA*, particulate methane monooxygenase; *mmoX*, methane monooxygenase; *aceA*, isocitrate lyase; *aceB*, malate synthase; *amyA*, alpha-amylase; *amyX*, amylopullulanase; *apu*, amylopullulanase; *cda*, cyclomaltodextrin dextrin-hydrolase; gluco., glucoamylase; isop., isopullulanase; *nplT*, neopullulanase; *pulA*, pullulanase; b-ara., bacterial arabinofuranosidase; f-ara., fungal arabinofuranosidase; mann., mannanase; *xylA*, xylose isomerase; xylan., xylananse; CDH, cellobiose dehydrogenase; cello., cellobiase; endogl., endoglucanase; exogl., exoglucanase; pect., pectinase; *assA*, alkylsuccinate synthase; *limEH*, limonene epoxide hydrolase; *lmo*, limonene monooxygenase; *vanA*, vanillate monooxygenase; *vdh*, vanillin dehydrogenase; acetylgl., acetylglucosaminidase; endoch., endochitinase; exoch., exochitinase; *glx*, glyoxal oxidase; *lip*, lignin peroxidase; *mnp*, manganese peroxidase; *lcc*, phenol oxidase.

A total of 2,695 C cycling genes were detected among all biofilm samples, most of which were associated with autotrophic C fixation (22.4%) and various C degradation pathways (72. 2%). The close grouping of most samples in an MDS ordination indicated that most samples had relatively similar proportions of C cycling gene families regardless of catchment type (Figs 2a and 3, S2 Fig). Samples from an urban stream and two rural waterways were widely scattered, however, suggesting that these streams had divergent proportions of carbon cycling gene families. SIMPER analysis indicated that variation in proportions of C degradation and autotrophic C fixation pathway genes contributed most to these differences. In particular, samples from both the urban stream (Oakley) and a rural river (Hoteo) had much lower proportions of glyoxylate pathway genes than biofilm from all other streams (Fig 3). The rural Hoteo river also had the lowest proportion of C fixation genes but the highest proportions of phenol oxidase, arabinofuranosidase, and starch degradation genes. A large number of acetogenesis, methane metabolism, and C degradation genes (other than glyoxylate pathway and aromatics degradation genes) were only detected in biofilm from the urban stream (Oakley).

Several C cycling gene families had contrasting average proportions in native forest stream samples compared to exotic forest stream samples. For example, the proportions of PCC carbon fixation genes, CDH genes for cellulose degradation, *xylA* genes for hemicellulose degradation, and *lcc* (phenol oxidase) genes for lignin degradation were higher in exotic forest stream samples than native forest stream samples (S2 Fig). Conversely, the proportion of acetylglucosaminidase genes (chitin degradation) was higher in native forest stream samples than in exotic forest stream samples.

### Nitrogen cycling genes

**Fig 4:**
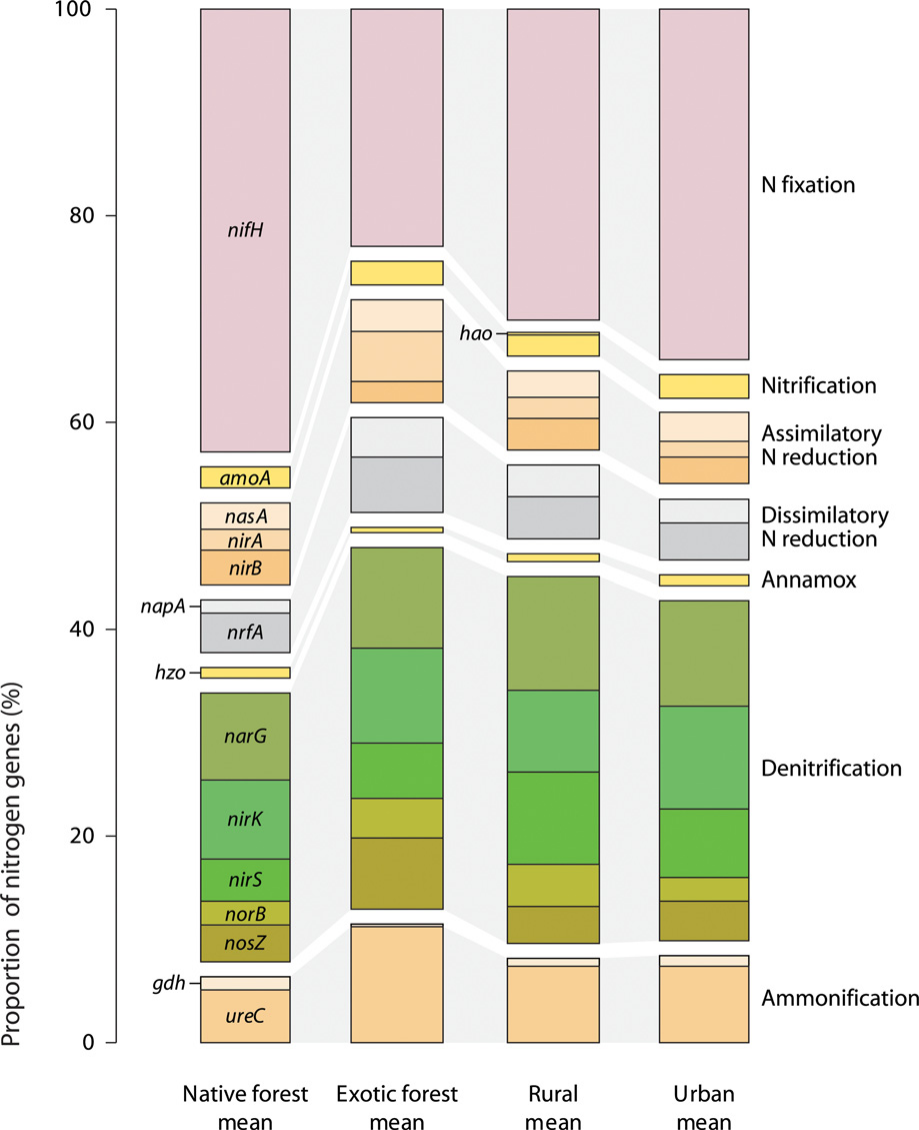
Average relative proportions of N cycling gene families and categories detected in microbial biofilm samples from native forest, exotic forest, rural and urban streams. *nifH*, dinitrogenase reductase; *hao*, hydroxylamine oxidoreductas; *amoA*, ammonia monooxygenase; *nasA*, nitrate reductase; *nirA* and *nirB*, nitrite reductases; *napA*, nitrate reductase; *nrfA*, c-type cytochrome nitrite reductase; *hzo*, hydrazine oxidoreductase; *narG*, nitrate reductase; *nirK* and *nirS*, nitrite reductases; *norB*, nitric oxide reductase; *nosZ*, nitrous oxide reductase; *gdh*, glutamate dehydrogenase; *ureC*, urease.

There were 1,606 nitrogen cycle-related genes detected in stream biofilm samples, most of which were dinitrogen reductase (*nifH)* genes for nitrogen fixation (561 in total), and various denitrification genes (641 in total). ANOSIM analysis found clear evidence of differences between proportions of N cycling genes detected in samples from exotic forest and native forest catchments, exotic forest and urban catchments, rural and urban catchments, and native forest and rural catchments (Table 3). Similarly, MDS ordination of samples based on proportions of N cycling genes showed a distinct cluster of urban and native forest samples, and a looser group of exotic forest and most rural samples (Fig 2b). Samples from streams with the most divergent proportions of C cycling genes (Hoteo, Ngakaroa, and Oakley) had comparatively more similar N cycling genes. SIMPER analysis indicated that differing proportions of *nifH* and denitrification genes contributed the most to the differences between catchment types. The average proportion of *nifH* genes in native forest and urban stream samples was 46% and 37% respectively, compared with 33% in rural samples and 25% in exotic forest samples (Fig 4 and S3 Fig). Conversely, the average total proportion of five denitrification genes was 29% and 36% respectively in native forest samples and urban samples, and 39% in both exotic forest and rural samples. Of the denitrification genes, rural stream samples had the highest (9.7%) and native forest stream samples the lowest (4.7%) average proportions of *nirS* genes (Fig 4 and S3 Fig). Exotic forest stream samples had relatively more *norB* genes than native forest and urban streams, and the highest proportion of *nosZ* genes (7%). The proportion of ammonification gene *ureC* was highest in exotic forest stream samples (12.3%) and lowest in native forest samples (5.6%). Exotic forest samples also had the highest average proportions of assimilatory (*nirA)* and dissimilatory (*napA* and *nrfA*) nitrogen reduction genes.

### Sulphur, phosphorus and energy metabolism genes

**Fig 5:**
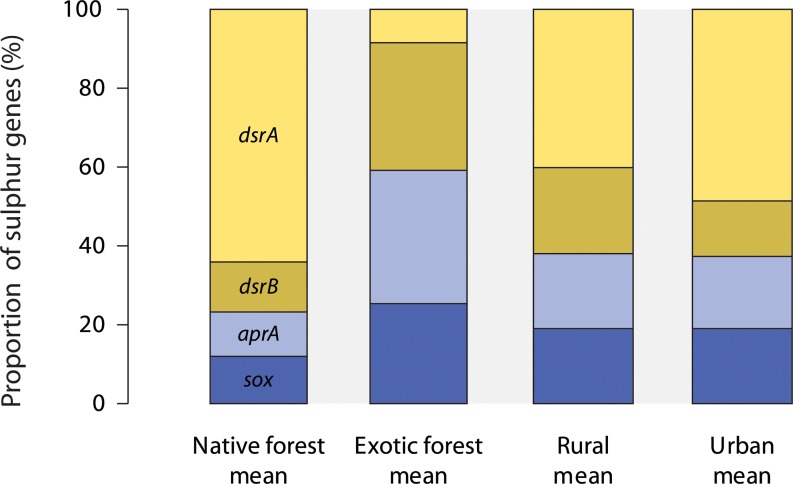
Average relative proportions of S cycling gene families and categories detected in microbial biofilm samples from native forest, exotic forest, rural and urban streams. *dsrA* and *dsrB*, sulphite reductases; *aprA*, dissimilatory adenosine-5’ phosphosulphate reductase; *sox*, sulphite oxidase.

There were 582 S cycling genes, belonging to four gene families, detected in biofilm samples. Most of these were genes for sulphite reductases (*dsrA* and *dsrB*), while sulphite oxidase (*sox*) and dissimilatory adenosine-5’-phosphosulphate reductase (*aprA*) genes occurred at lower frequencies. The proportions of S cycling genes was different in native forest and urban samples compared to exotic forest and rural samples (Table 3). This was attributed mainly to contrasting proportions of sulphite reductase genes *dsrA* and *dsrB*, with the former accounting for 64% and 48% of S cycling genes in native forest and urban stream samples, compared to 40% in rural stream samples and just 9% in exotic forest stream samples. Conversely, *dsrB* genes accounted for 12% and 18% of S cycling genes respectively in native forest and urban stream samples, compared to 21% and 32% in rural and exotic forest stream samples (Fig 5 and S4 Fig). Exotic forest stream samples had a higher average proportion of dissimilatory adenosine-5’-phosphosulphate reductase (*aprA)* genes (34%) than other stream types (11–19%). Exotic forest streams also had the highest average proportion of sulphite oxidase (*sox*) genes (26%) and native forest stream samples had the lowest (12%).

A total of 247 P cycling genes (in three gene families) and 241 energy metabolism genes (in two gene families) were detected in biofilm samples. Most of the P cycling genes (about 71%) were for hydrolysis of inorganic polyphosphate (*ppx*), followed by genes for polyphosphate biosynthesis (*ppk*, 27%), and a very low number of phytase genes. On average 65% of the energy metabolism genes detected in biofilm samples were c-type cytochromes and 35% were hydrogenases. There was no evidence of differences in P cycling or energy metabolism gene composition between different types of stream (S4 Fig).

### Taxonomic composition of biofilm assemblages

Most of the nutrient cycling/energy metabolism genes detected in biofilm samples were of bacterial origin (85%, 22 phyla and 39 classes), with smaller proportions of archaeal genes (4%, 2 phyla and 6 classes) and eukaryote genes (10%, 3 phyla and 13 classes, mainly fungi) (Table 4). Fewer taxa were present among the 638 *gyrB* phylogenetic marker genes (15 phyla and 27 classes of Bacteria, and 1 phylum and 4 classes of Archaea; Table 4). Genes from Proteobacteria and unclassified bacteria were most common among nutrient/energy genes, followed by Actinobacteria, Firmicutes, and Ascomycota (S3 Table). There was a higher proportion of Proteobacteria and Tenericutes among *gyrB* genes compared to nutrient energy genes, and a lower proportion of unclassified Bacteria. Oakley Creek biofilm (urban) had the highest proportion of *gyrB* genes, including several taxa that were undetected in most other samples (such as Chlamydiae, Chlorobia, Chloroflexi, and several classes of Cyanobacteria and Archaea). As for nutrient cycling/energy metabolism genes, about half (388) of *gyrB* genes were detected in only one sample, while just 25 *gyrB* genes (from 18 different genera) were detected in ≥ 15 samples.

**Table 4:** Numbers of different phyla, classes, orders, families, and genera represented among nutrition/energy genes and *gyrB* genes in stream biofilms.

	Domain	Phyla	Classes	Orders	Families	Genera
Nutrition/energy genes	Archaea	2	6	16	15	41
	Bacteria	22	39	69	148	413
	Eukaryota	3	13	32	64	118
*gyrB* genes	Archaea	1	4	5	7	9
	Bacteria	15	27	59	122	252

An MDS ordination based on the proportions of genera represented among *gyrB* genes (Fig 2c) was very similar to that based on all nutrient cycling/energy metabolism genes (Fig 1a). Comparisons of Bray-Curtis sample similarity matrices based on proportions of nutrition/energy gene families with the similarity matrix based on *gyrB* genera using RELATE indicated that there were statistically significant matches for most gene categories. The similarity matrix based on C cycling gene families was most similar to the *gyrB*–based similarity matrix (*ρ* = 0.655, p = 0.001), followed by N cycling gene families (*ρ* = 0.418, p = 0.004), S cycling gene families (*ρ* = 0.321, p = 0.01), and energy metabolism gene families (*ρ* = 0.413, p = 0.004), suggesting a link between taxonomic composition and functional gene composition within these categories. The match with a similarity matrix based on P cycling gene families was not significant.

## Discussion

A growing number of studies have considered the taxonomic composition of microbial communities in aquatic environments, enabled by advances in high-throughput molecular analysis techniques [28, 36–38]. The ecologically-relevant functional composition of microbial communities is less well understood, but improving knowledge of these aspects is important for increasing our understanding of the biogeochemical processes which maintain the biosphere. Our results demonstrate the presence of a diverse range of nutrient cycling and energy metabolism genes in stream biofilms, consistent with the possibility that these communities contribute significantly to key biogeochemical processes in streams.

This study included streams representing a wide variety of ecological states, from near-pristine waterways in forested catchments to highly modified and degraded waterways in highly urbanized catchments, with documented differences in nutrient and pollution levels and shading (Table 1 and S1 Table). In light of these environmental differences, the observed broad similarity of biofilm functional gene composition among different streams is perhaps surprising. Similar functional gene assemblages have been observed in GeoChip-based investigations of uranium-contaminated groundwater [39], healthy and diseased corals [40], and acid mine drainage [41]. This suggests that natural microbial communities may develop broadly similar functional gene composition and metabolic potential despite varied environmental conditions, and that environmental degradation may have only a limited impact upon microbial metabolic potential. The finding that most genes from specific taxa were detected only in one or a few samples suggests either a high degree of taxonomic divergence between streams or, more probably, that our analysis has sampled only a very small proportion of the total microbial functional gene diversity present in stream biofilms. Nonetheless, that most gene families were present in most samples suggests the existence of functional gene redundancy among stream biofilm communities. This may indicate that biofilm communities include a reservoir of microbes with the potential to restore or repair perturbed ecological processes which may arise in degraded streams.

Microbial community composition can be influenced by environmental conditions [28, 42], and changes in microbial community composition may affect ecological functions [21, 22], such as C degradation [43] and denitrification [18]. However, other studies report no links between stream microbial community composition and the potential activity of a suite of C-, N- and P-related enzymes [44]. A fine-grained examination of our results revealed that although overall biofilm functional gene composition was similar among streams, differences were evident in the relative abundance and occurrence of specific functional genes, clearly indicating potential for enzymatic functions to vary in biofilms from different streams. Of course, the presence of functional genes does not necessarily mean that those genes are actively expressed or contributing to biogeochemical processes. To confirm whether this is the case, it may be necessary to collect parallel data on gene occurrence, gene expression and enzymatic activity in stream biofilms at different time points.

Factors previously identified as influencing stream benthic bacterial community composition include pH and conductivity [45], carbon and nutrients [46], and broad-scale land use [28]. This is consistent with our results, in which in-stream N concentration and pH, along with water temperature, shade and catchment land use, were correlated with the similarity of functional gene assemblages according to MDS ordination (Fig 1b and S2 Table). Our results suggest that the reduced N levels, increased shade and reduced water temperatures observed in exotic forest streams compared to urban streams contribute to the functional gene assemblage differences observed between these types of streams (Fig 1b). Functional gene assemblages in urban streams with moderate levels of N, shade and temperature (Lucas, Otara, Oteha) appear to have intermediate similarity to biofilm from exotic forest streams, while samples from the urban streams with the least shade, the highest temperatures and the lowest water quality (Omaru, Pakuranga and Puhinui) have more divergent functional gene assemblages. This suggests increasing levels of degradation may cause increasing divergence of biofilm functional gene composition from that observed in non-degraded stream ecosystems.

Three rural streams with partially forested catchments (Okura, Makarau, and Rangitopuni) also seem to have relatively similar biofilm functional gene assemblages to exotic forest streams, but biofilm samples from the native forest streams do not follow this pattern. In particular, both the functional gene composition and *gyrB* taxonomic composition of biofilm from Cascade stream, the most pristine environment in this study, was similar to biofilm from urban streams. A previous investigation found that bacterial biofilm community composition in Cascade Stream was most similar to that in urban streams including considerably degraded Omaru Stream [42], which is consistent with the present study. Cascade Stream substrates have copper concentrations that are higher than in other forested and most rural streams and comparable to many degraded urban streams [47]. Copper and other metals accumulate in stream biofilms [12] and have significant effects on biofilm communities [12, 48] and microbial gene composition [49]. This factor may therefore contribute to the high similarity observed between Cascade Stream and urban stream biofilms. The source of copper in Cascade Stream is unclear, but may be linked to the underlying volcanic geology of this catchment [50]. The functional gene composition of biofilm from urban Lucas Creek appears very similar to that in Cascade Stream biofilm, however, yet Lucas Creek has low levels of copper. It thus appears that biofilm bacterial communities and functional genes may be driven towards similar composition by (or despite) many contrasting environmental factors present in urban and native forest streams.

While differences between exotic forest and urban stream functional gene assemblages can be readily ascribed to obvious vegetation cover and built environment differences, divergent factors between exotic forest catchments compared to native forest catchments are more subtle. Clear-felling and replanting of pine forest may cause drastic disturbances such as reduced surface water retention, elevated sediment loads entering streams, loss of leaf litter inputs and increased sunlight exposure, potentially altering the balance of biogeochemical cycles within streams on a multi-decadal basis. A variety of differences have been observed between streams in catchments planted with pine forest compared with streams in native broadleaf forest catchments, including increased levels of sediment, turbidity, dissolved organic C, N, and woody debris in pine forest streams [51], and altered benthic invertebrate communities and in-stream decomposition dynamics [52]. Pine needles are tough and less easily retained in waterways, with protective biochemical compounds and high C:N ratios, thus representing a less labile and lower quality nutrient resource in streams than litter from typical broadleaf species [53, 54]. Additionally, benthic microbial enzyme activity may vary between native forest and pine forest streams, presumably in response to altered dissolved organic C supply [8]. This suggests that altered functional gene potential in pine forest and native forest streams is likely, and this is supported by our results.

Biofilm from rural streams showed the most variation in number and composition of functional genes detected (Table 2 and Fig 1). This may be related to the mix of land use activities represented within rural catchments. While pastoral agricultural activity has well-established and long-acting impacts upon rural waterways [2], rural catchments also contain varying proportions of horticulture, native forest and exotic forest, with minor proportions of urban development (Table 1). This variation in rural land use patterns is likely to result in different inputs to rural streams and varied responses in rural stream biofilm communities. For example, streams in rural catchments with the highest proportion of forest cover had functional gene assemblages that were most similar to those detected in exotic forest streams. Rural Makarau Stream has one of the lowest in-stream N levels in this study, whereas rural Ngakaroa Stream has minimal forest cover but the highest proportion of horticultural land use in its catchment, and the highest in-stream N concentration (S1 Table). These contrasts are reflected by apparent functional gene differences between these streams (Figs 1b and 2b).

GeoChip analysis provides novel information about complex microbial communities and their potential functions, and represents a useful tool for investigating the dynamics of the microorganisms which underpin natural biogeochemical processes and cycles. Evidently, the analysis of genes using GeoChip is limited to the probes present on the chip, which is in turn constrained by the (steadily increasing) availability of robust sequence data. The number and range of probes present on the GeoChip has progressively increased on successive versions [24, 55, 56]. It nevertheless seems likely that this study has only “scratched the surface” of microbial functional gene diversity, despite the extent of data provided by GeoChip analysis. This is consistent with our observation that most individual genes were detected only in one or a few samples.

The observed patterns of functional gene composition and their relative gene abundance suggest the existence of functional gene redundancy in stream biofilms, as well as land-use related differences in stream microbial biofilm functional gene composition. This research raises many questions for further investigation. In particular, it is important to determine the degree to which functional gene expression and associated ecological processes in stream biofilms match the patterns of functional gene composition observed in this study. Our results also suggest hypotheses for further investigation regarding the occurrence and activity of particular functional genes or groups in relation to environmental parameters and taxonomic composition. As the costs of high-throughput sequencing steadily reduce, GeoChip analysis may provide a basis for metagenomic investigations of microbial biogeochemical functional genes of interest. Such investigations have the potential to provide insights into the regulation of biogeochemical cycling within streams, helping to clarify the role of microbial biofilm communities in maintaining the health and function of stream ecosystems.

## Supporting Information

S1 Fig Clustering of stream biofilm samples based on Bray-Curtis similarity of functional gene assemblages detected by triplicate Geochip analyses. (PDF)

S2 Fig Average numbers of genes detected in each C cycling gene family, expressed as proportions of the total number of C cycling genes detected in biofilm samples from native forest, exotic forest, rural and urban streams. Error bars represent ± one standard deviation. Refer to Fig 3 legend for C cycling gene family details.
(PDF)

S3 Fig Average numbers of genes detected in each N cycling gene family, expressed as proportions of the total number of N cycling genes detected in biofilm samples from native forest, exotic forest, rural and urban streams. Error bars represent ± one standard deviation. Refer to Fig 4 legend for N cycling gene family details.
(PDF)

S4 Fig Average numbers of genes detected in each S cycling (a), P cycling (b), and energy metabolism (c) gene family, expressed as proportions of the total numbers of S cycling, P cycling or energy metabolism genes detected in biofilm samples from native forest, exotic forest, rural and urban stream biofilm samples. Error bars represent ± one standard deviation. Refer to Fig 5 legend for S cycling gene family details. P cycling gene families: *ppk*, polyphosphate kinase; *ppx*, exopolyphosphatase.
(PDF)

S1 Table Chemical water quality attributes and biofilm metal concentrations of the streams sampled for this study. (PDF)

S2 Table Correlations between environmental parameters and nutrition/energy metabolism gene assemblages detected in stream biofilm samples. (PDF)

S3 Table Taxonomic composition of nutrition/energy metabolism genes and *gyrB* genes detected in stream biofilm samples. (PDF)

S4 Table Stream biofilm Geochip 3.0 probe hybridization data. (ZIP)
