# Supplementary figures and images for: Functional Gene Composition, Diversity and Redundancy in Microbial Stream Biofilm Communities
Source: PLoS One. 2015 Apr 7;10(4):e0123179. doi: 10.1371/journal.pone.0123179 (PMC4388685; doi:10.1371/journal.pone.0123179)

Similarity (%)

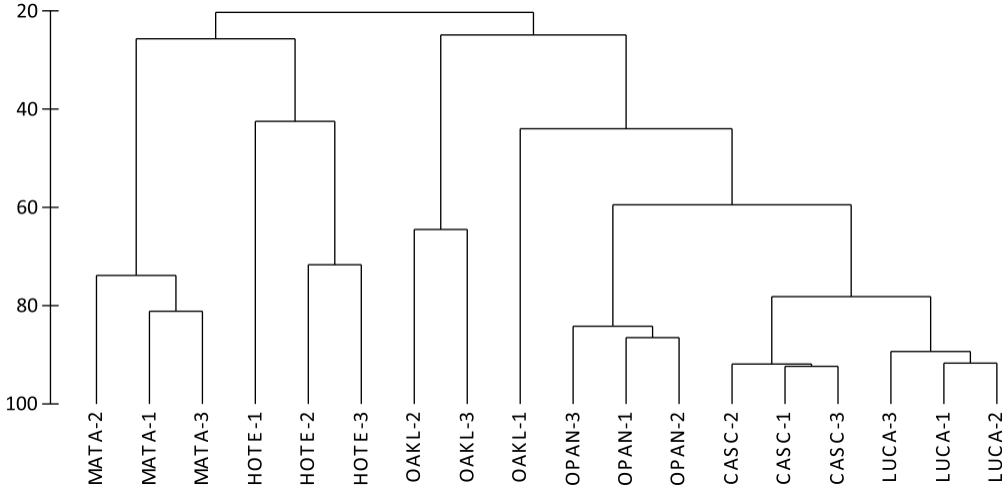

Supplement: S1 Fig — (PDF) [file pone.0123179.s001.pdf]

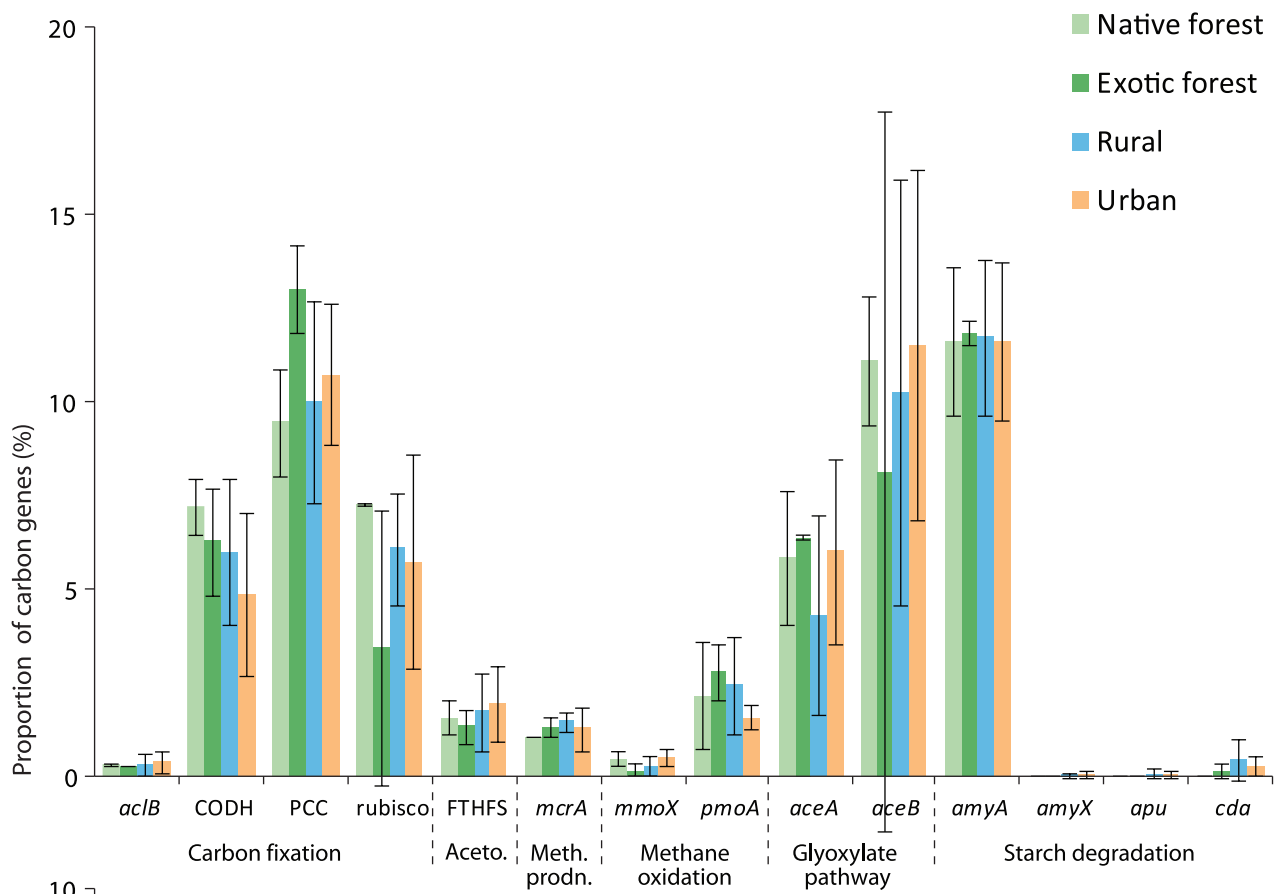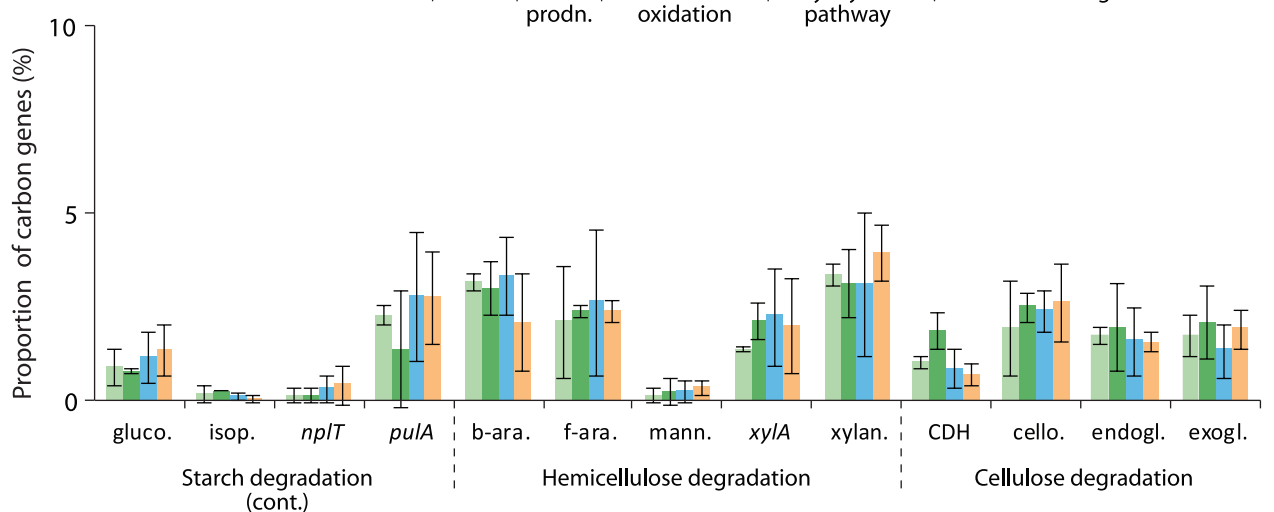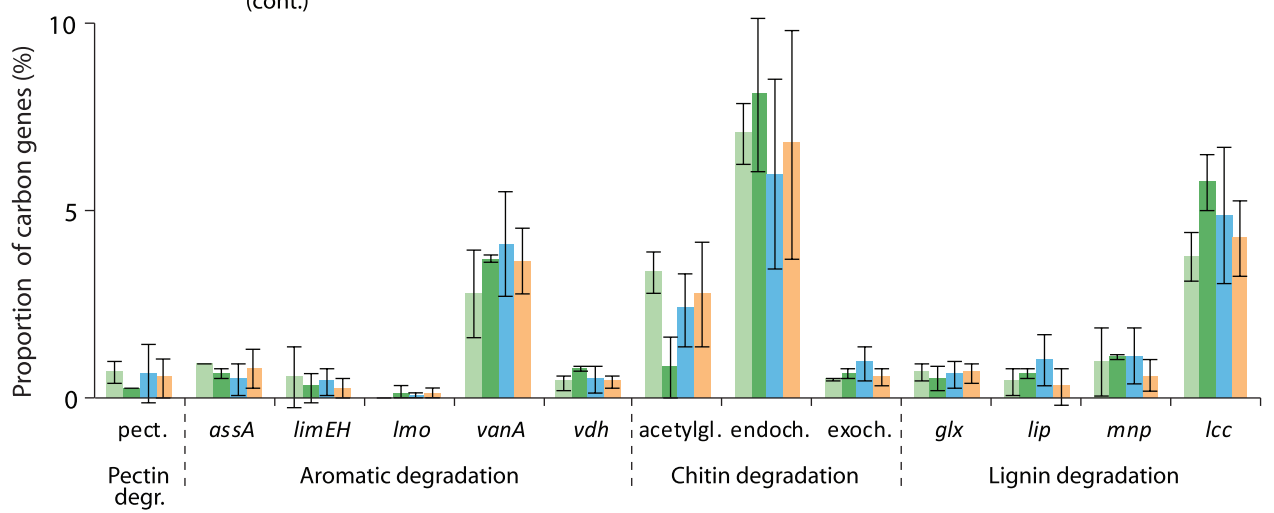

Supplement: S2 Fig — Error bars represent ± one standard deviation. Refer to Fig 3 legend for C cycling gene family details. (PDF) [file pone.0123179.s002.pdf]

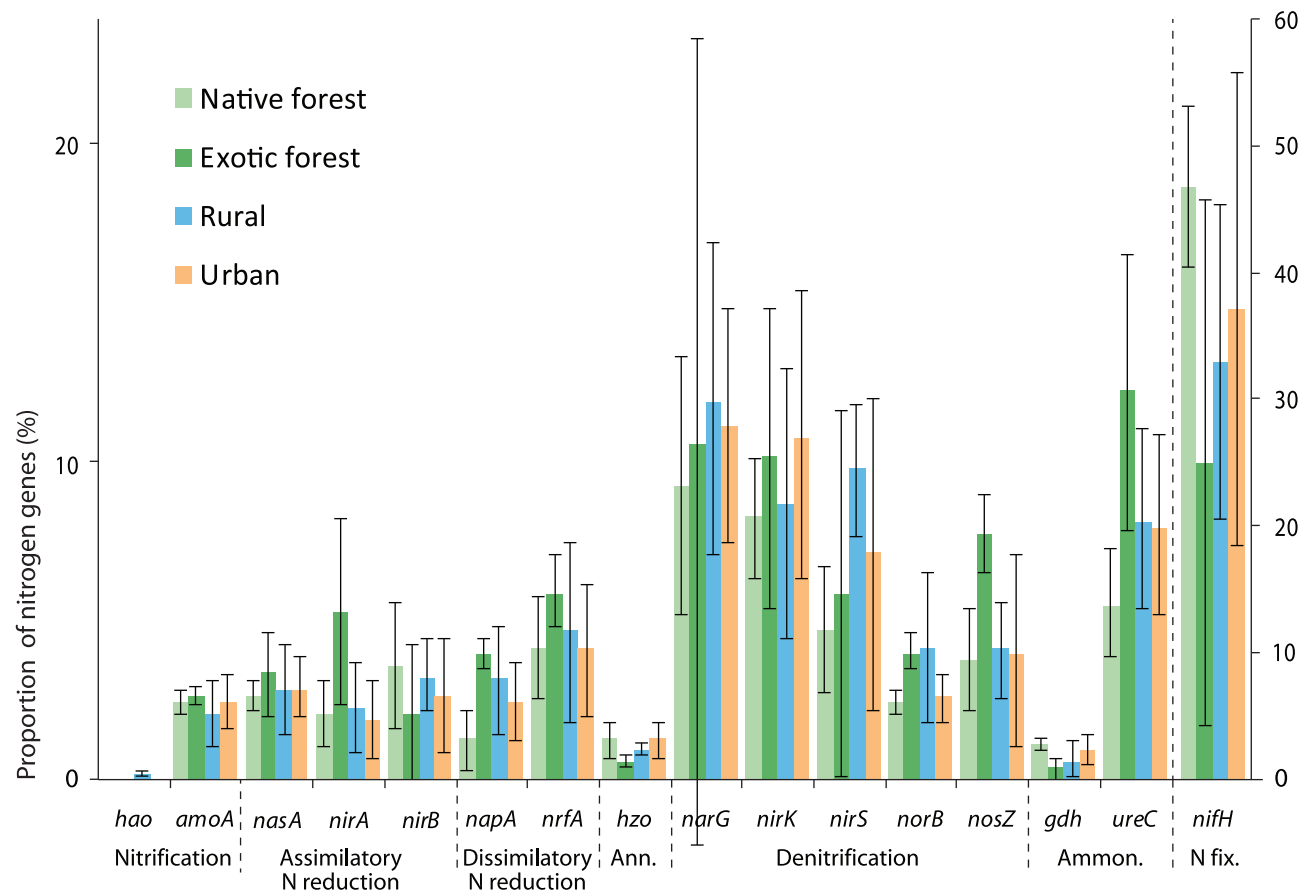

Supplement: S3 Fig — Error bars represent ± one standard deviation. Refer to Fig 4 legend for N cycling gene family details. (PDF) [file pone.0123179.s003.pdf]

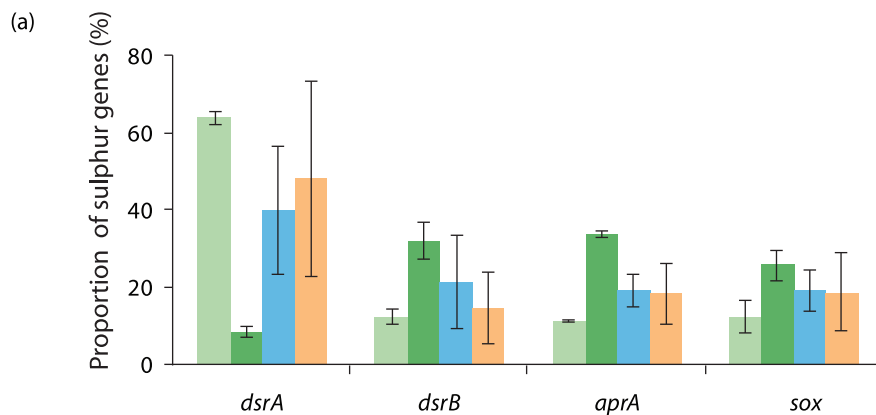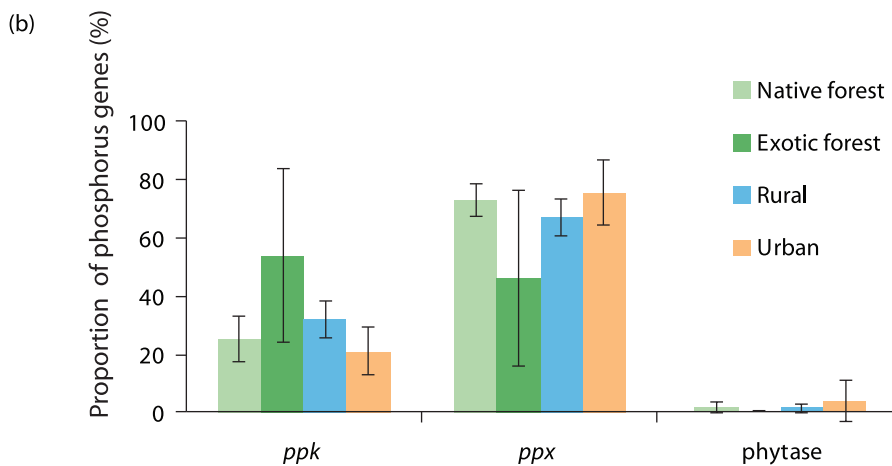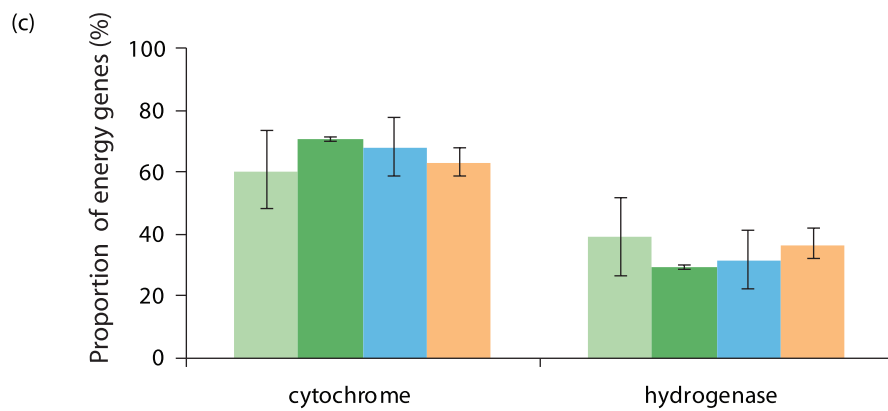

Supplement: S4 Fig — Error bars represent ± one standard deviation. Refer to Fig 5 legend for S cycling gene family details. P cycling gene families: ppk, polyphosphate kinase; ppx, exopolyphosphatase. (PDF) [file pone.0123179.s004.pdf]
