# Supplementary material for: Functional Gene Composition, Diversity and Redundancy in Microbial Stream Biofilm Communities
Source: PLoS One. 2015 Apr 7;10(4):e0123179. doi: 10.1371/journal.pone.0123179 (PMC4388685; doi:10.1371/journal.pone.0123179)
Supplement: S1 Table — (PDF) [file pone.0123179.s005.pdf]

S1 Table: Chemical water quality attributes and biofilm metal concentrations of the streams sampled for this study.

Stream	Temperature. (º C) [1]	Conductivity (mS/cm) [1]	pH [1]	Turbidity (NTU) [1]	Dissolved Oxygen (ppm) [1]	Ammoniacal Nitrogen (mg/L) [1]	Kjeldahl Nitrogen (mg/L) [1]	Total Nitrogen (mg/L) [1]	Soluble Phosphorus (mg/L) [1]	Total Phosphorus (mg/L) [1]	Biofilm Copper (mg/kg) [2]	Biofilm Lead (mg/kg) [2]	Biofilm Zinc (mg/kg) [2]
CASC	13.4 (1.74)	0.137 (0.042)	7.4 (0.3)	9.2 (9.3)	10.1 (1.9)	0.016 (0.022)	0.264 (0.27)	0.282 (0.274)	0.017 (0.003)	0.036 (0.02)	54.1 (19.7)	2.2 (0.1)	177.9 (39.5)
OPAN	13.5 (2.14)	0.155 (0.019)	7.3 (0.5)	12.6 (9.3)	10.1 (0.9)	0.016 (0.013)	0.370 (0.190)	0.475 (0.220)	0.011 (0.006)	0.036 (0.021)	34.1 (4.6)	6.1 (3.8)	245.7 (74.9)
MAHU	14.4 (2.2)	0.221 (0.036)	7.4 (0.2)	14.4 (18.1)	10.0 (1.8)	0.025 (0.014)	0.345 (0.214)	0.409 (0.210)	0.007 (0.002)	0.036 (0.036)	21.6 (2.6)	3.4 (0.6)	74.8 (1.9)
RIVE	13.6 (2.4)	0.158 (0.036)	6.3 (0.4)	11.3 (5.7)	8.5 (1.1)	0.011 (0.002)	0.312 (0.081)	0.383 (0.081)	0.009 (0.005)	0.020 (0.003)			
MATA	15.7 (1.9)	0.226 (0.068)	7.2 (0.2)	6.8 (4.0)	8.4 (1.5)	0.016 (0.009)	0.351 (0.135)	0.394 (0.140)	0.017 (0.011)	0.039 (0.013)	62.3 (15)	9.8 (1.3)	276.9 (85.9)
OKUR	13.8 (1.8)	0.157 (0.061)	7.3 (0.2)	30.0 (13.0)	8.8 (1.6)	0.027 (0.018)	0.577 (0.234)	0.830 (0.335)	0.023 (0.017)	0.072 (0.038)			
KUME	15.7 (1.9)	0.133 (0.056)	7.0 (0.5)	15. 1 (10.3)	8.4 (1.4)	0.021 (0.015)	0.533 (0.193)	0.800 (0.229)	0.021 (0.006)	0.068 (0.031)	62.7 (5.6)	31.4 (8.5)	358.6 (52.0)
MAKA	15.3 (3.0)	0.218 (0.057)	7.5 (0.2)	9.3 (2.1)	10.4 (1.3)	0.019 (0.018)	0.317 (0.096)	0.397 (0.176)	0.012 (0.003)	0.033 (0.009)			
RANG	15.6 (2.0)	0.195 (0.017)	7.2 (0.1)	7.9 (2.8)	7.7 (1.2)	0.024 (0.007)	0.469 (0.082)	0.615 (0.101)	0.018 (0.003)	0.063 (0.007)	24.3 (3.9)	6 (0.3)	183.1 (43.9)
HOTE	16.8 (1.9)	0.136 (0.015)	7.4 (0.2)	11.6 (6.3)	8.8 (0.5)	0.016 (0.008)	0.319 (0.102)	0.560 (0.170)	0.021 (0.008)	0.064 (0.015)	25.1 (1.3)	7.1 (0.5)	123.4 (38.5)
NGAK	14.9 (2.1)	0.244 (0.043)	7.3 (0.3)	5.1 (3.7)	9.3 (2.2)	0.013 (0.008)	0.360 (0.128)	2.712 (0.561)	0.008 (0.002)	0.025 (0.017)	18.7	5.2	84.4
LUCA	15 (1.6)	0.194 (0.038)	7.2 (0.2)	15.4 (9.1)	8.7 (1.8)	0.029 (0.017)	0.424 (0.144)	0.599 (0.163)	0.013 (0.004)	0.044 (0.022)	24.4 (2.2)	17.3 (16.6)	182.2 (19.2)
OTAR	16.2 (2.3)	0.216 (0.033)	7.3 (0.4)	12.4 (9.1)	8.8 (2.6)	0.036 (0.011)	0.501 (0.100)	0.834 (0.285)	0.033 (0.033)	0.083 (0.033)	38 (8.5)	48.4 (5.9)	1064.1 (63.3)
PUHI	17.8 (3.1)	0.202 (0.039)	7.8 (0.6)	9.3 (6.0)	11.6 (2.1)	0.028 (0.012)	0.473 (0.149)	0.964 (0.259)	0.021 (0.012)	0.067 (0.029)	44.0 (3.1)	7.0 (2.6)	591.2 (157.8)
OTEH	15.1 (1.6)	4.506 (0.049)	7.1 (0.6)	10.9 (7.3)	7. 9 (3.1)	0.054 (0.035)	0.547 (0.170)	0.849 (0.242)	0.015 (0.004)	0.052 (0.015)	228.8 (45.7)	39.6 (7.5)	310.9 (84)
OMAR	17.4 (1.8)	0.248 (12.55)	7.5 (0.2)	7.2 (1.7)	7.3 (2.1)	0.114 (0.095)	0.580 (0.273)	1.132 (0.442)	0.022 (0.008)	0.072 (0.033)	125.5 (23.7)	54.2 (19.9)	3411.7 (486)
OAKL	15.7 (1.9)	0.278 (0.118)	7.4 (0.4)	7.5 (7.0)	9.2 (1.8)	0.028 (0.014)	0.473 (0.196)	1.762 (0.433)	0.025 (0.009)	0.072 (0.036)	63.1 (10.7)	69.8 (17.8)	1458.9 (579.4)
PAKU	19.3 (2.0)	0.455 (0.027)	7.4 (0.3)	9.4 (2.7)	11.2 (1.2)	0.059 (0.023)	0.547 (0.185)	1.446 (0.366)	0.016 (0.013)	0.055 (0.020)	41.8 (5.8)	9.0 (1.2)	957.9 (108.6)

[1] Chemical water quality values are means of measurements taken in early-mid October, November and December of 2007-2009 (standard deviations in parentheses). Data for all streams except for HOTE and RANG are from the River Water Quality Monitoring Programme operated by the Auckland Council (Neale 2010a; Neale 2010b; Neale 2009; Data available at http://maps.aucklandcouncil.govt.nz/aucklandcouncilviewer/). Data for HOTE and RANG are from the National River Water Quality Network operated by the National Institute of Water and Atmospheric Research (Graham Bryers, NIWA, New Zealand, http://www.niwa.co.nz). NTU denotes nephelometric turbidity units. [2] Biofilm metal values are means and standard deviations based on dry weight of biofilm samples collected in January 2008 (Ancion 2010).
