# Supplementary material for: Functional Gene Composition, Diversity and Redundancy in Microbial Stream Biofilm Communities
Source: PLoS One. 2015 Apr 7;10(4):e0123179. doi: 10.1371/journal.pone.0123179 (PMC4388685; doi:10.1371/journal.pone.0123179)
Supplement: S2 Table — (PDF) [file pone.0123179.s006.pdf]

S2 Table: Correlations between water quality parameters or catchment land use attributes with primary (horizontal) and secondary (vertical) axes of an MDS plot based on proportions of nutrition/energy gene families detected in stream biofilm samples (Fig 1b).

Parameter[1]	MDS axis 1		MDS axis 2		Shannon diversity of nutrition/energy genes[2]	
	Correlation	p-value	Correlation	p-value	Correlation	p-value
Temperature	0.21	0.461	-0.68	0.006*	-0.23	0.366
Conductivity	0.21	0.450	-0.21	0.445	-0.05	0.849
pH	0.27	0.322	-0.49	0.062	-0.26	0.317
Turbidity	-0.47	0.078	0.42	0.122	0.68	0.003*
Dissolved oxygen	0.11	0.696	-0.51	0.054	-0.16	0.533
Ammoniacal N	0.24	0.379	-0.41	0.129	0.03	0.897
Kjeldahl N	0.07	0.815	-0.23	0.402	0.32	0.205
Total N	0.77	0.001*	-0.21	0.454	-0.26	0.320
Soluble P	-0.07	0.791	-0.12	0.667	-0.01	0.973
Total P	-0.09	0.739	-0.21	0.460	0.03	0.897
Biofilm Lead	-0.07	0.838	-0.08	0.814	0.03	0.924
Biofilm Zinc	0.15	0.649	-0.3	0.335	-0.08	0.796
Biofilm Copper	0.03	0.915	-0.19	0.551	0.06	0.850
Shade at sample site	-0.19	0.544	0.66	0.020*	-0.18	0.567
Native forest %	0.07	0.798	0.1	0.730	0.03	0.911
Exotic forest %	-0.51	0.051	0.47	0.074	0.06	0.824
Horticulture %	0.64	0.010*	0.04	0.891	-0.16	0.541
Pastoral %	-0.04	0.890	0.11	0.684	0.07	0.799
Urban %	0.33	0.225	-0.63	0.011*	-0.11	0.685

[1]Water temperature was negatively correlated with native forest (-0.54, $p = 0.025$) and positively correlated with urban land use (0.61, $p = 0.009$); pH was negatively correlated with exotic forest (-0.54, $p = 0.024$); Total N was positively correlated with horticultural land use (0.73, $p = 0.001$). Biofilm lead and zinc were both positively correlated with urban land use (respectively 0.57, $p = 0.034$ and 0.60, $p = 0.023$). [2]Shannon diversity of *gyr*B genes and nutrient cycling/energy genes and were strongly correlated (0.90, $p < 0.001$), and correlations of each with environmental parameters showed the same trends.
