# Supplementary material for: Functional Gene Composition, Diversity and Redundancy in Microbial Stream Biofilm Communities
Source: PLoS One. 2015 Apr 7;10(4):e0123179. doi: 10.1371/journal.pone.0123179 (PMC4388685; doi:10.1371/journal.pone.0123179)
Supplement: S3 Table — (PDF) [file pone.0123179.s007.pdf]

S3 Table: Taxonomic composition (%) of nutrition/energy metabolism genes, and *gyrB* phylogenetic marker genes detected in stream biofilms.

Nutrition/energy genes

		Stream																
Domain	Phylum	CASC	OPAN	MAHF	RIVE	NGAK	OKUR	RANG	HOTE	KUME	MAKA	OTKH	LUCA	PUHI	OTET	OMAR	OAKL	PAKB
	Crenarchaeota	1.7	1.4	0.9	1.3	1.8	0.8	1.0	1.3	1.3	0.9	1.2	1.4	1.5	1.4	1.4	0.5	1.7
Archaea	Euryarchaeota	3.4	2.3	2.3	2.0	2.3	2.2	1.9	1.9	2.6	2.6	2.8	3.3	2.6	2.1	2.7	2.9	2.8
	Unclassified Archaea	0.2	0.4	0.3	0.1	0.3	0.1	0.2	0.2	0.3	0.6	0.2	0.2	0.2	0.3	0.2	0.7	0.4
	Acidobacteria	0.5	0.5	0.6	0.8	1.2	0.5	0.8	0.6	0.6	0.7	0.6	0.5	0.5	0.7	0.4	0.7	0.9
	Actinobacteria	6.0	4.8	7.6	5.9	5.8	6.9	7.4	3.8	5.7	6.7	7.5	6.3	5.4	6.0	5.8	7.8	5.4
	Aquificae	0.1	0.2	0.1	0.0	0.0	0.1	0.1	0.2	0.1	0.1	0.1	0.1	0.2	0.1	0.1	0.2	0.0
	Bacteroidetes	2.0	1.2	1.4	1.3	1.8	1.6	1.2	3.4	2.4	1.3	1.4	1.9	1.7	1.4	2.1	2.8	2.4
	Chlamydiae	0.2	0.4	0.2	0.2	0.6	0.1	0.2	0.6	0.2	0.1	0.2	0.2	0.1	0.3	0.1	0.2	0.2
	Chlorobi	1.2	1.2	0.9	0.7	1.2	0.8	1.0	0.8	0.8	1.2	1.0	1.1	1.1	1.0	1.4	0.8	1.3
	Chloroflexi	1.5	1.4	1.8	1.6	0.6	1.6	1.9	1.9	1.1	2.0	1.2	1.2	1.1	1.0	1.2	1.0	1.1
	Cyanobacteria	3.4	1.8	1.5	1.9	2.0	2.0	2.2	1.9	2.3	2.5	2.9	3.4	2.2	2.4	3.2	2.7	2.0
	Deinococcus-Thermus	0.8	0.7	0.8	0.3	0.9	0.5	0.6	0.2	0.6	0.6	0.6	0.8	0.6	0.7	0.6	0.5	0.4
	Dictyoglomi	0.0	0.0	0.0	0.0	0.0	0.0	0.0	0.0	0.0	0.0	0.0	0.0	0.0	0.0	0.0	0.1	0.0
	Firmicutes	8.0	7.3	6.7	6.0	7.6	5.8	5.9	13.0	8.0	7.1	6.4	7.9	8.4	7.8	9.0	8.9	9.1
Bacteria	Fusobacteria	0.0	0.0	0.0	0.1	0.0	0.0	0.1	0.0	0.0	0.0	0.0	0.0	0.1	0.0	0.0	0.0	0.0
	Lentisphaerae	0.0	0.0	0.1	0.1	0.0	0.0	0.1	0.0	0.1	0.0	0.0	0.0	0.1	0.0	0.0	0.0	0.0
	Nitrospirae	0.1	0.0	0.0	0.0	0.0	0.0	0.0	0.0	0.0	0.0	0.0	0.1	0.0	0.0	0.0	0.0	0.0
	Planctomycetes	0.7	0.7	0.4	0.3	1.2	0.5	0.7	0.4	0.3	0.4	0.6	0.5	0.5	0.7	0.6	0.4	0.9
	Proteobacteria	30.3	35.3	34.8	37.1	32.2	37.0	34.1	33.3	30.3	33.0	34.3	31.2	31.9	33.9	31.7	33.4	32.4
	Spirochaetes	0.5	0.4	0.1	0.2	0.3	0.2	0.2	0.0	0.3	0.3	0.4	0.5	0.1	0.1	0.6	0.1	0.2
	Synergistetes	0.1	0.2	0.0	0.0	0.0	0.0	0.0	0.0	0.1	0.0	0.0	0.0	0.0	0.0	0.0	0.0	0.0
	Tenericutes	0.0	0.0	0.0	0.0	0.0	0.1	0.0	0.0	0.0	0.0	0.0	0.0	0.1	0.0	0.0	0.2	0.2
	Thermodesulfobacteria	0.0	0.0	0.2	0.1	0.0	0.1	0.1	0.2	0.2	0.0	0.0	0.0	0.1	0.1	0.0	0.0	0.0
	Thermotogae	0.3	0.2	0.2	0.2	0.3	0.2	0.2	0.6	0.3	0.1	0.4	0.2	0.2	0.3	0.2	0.3	0.2
	Unclassified Bacteria	31.2	30.8	29.0	30.7	31.6	30.5	31.1	17.4	32.3	29.1	30.2	30.7	32.2	31.2	30.2	21.9	29.8
	Verrucomicrobia	0.3	0.7	0.4	0.9	0.6	0.5	0.5	0.6	0.3	0.9	0.6	0.4	0.5	0.5	0.5	0.2	0.7
Prokaryota	Unclassified Prokaryote	0.0	0.2	0.1	0.1	0.3	0.1	0.1	0.0	0.2	0.3	0.2	0.1	0.1	0.1	0.1	0.1	0.4
	Ascomycota	4.3	3.9	5.7	4.9	5.0	4.0	4.8	10.2	6.2	5.1	4.1	4.7	5.2	4.6	4.7	8.3	4.6
Eukaryota	Basidiomycota	1.6	2.5	2.7	2.0	2.3	2.5	2.2	5.1	2.4	2.6	2.2	1.6	1.8	1.7	1.5	4.0	1.7
	Mucorales	0.0	0.0	0.0	0.0	0.0	0.0	0.0	0.0	0.0	0.0	0.0	0.0	0.0	0.0	0.0	0.1	0.0

	Neocallimastigomycota	0.0	0.2	0.1	0.1	0.0	0.1	0.1	0.2	0.0	0.0	0.1	0.0	0.1	0.1	0.1	0.2	0.0
	Unclassified Fungi	0.2	0.7	0.5	0.4	0.3	0.5	0.5	1.9	0.5	1.2	0.4	0.4	0.6	0.5	0.6	0.8	0.4
	Trypanosomatidae	0.0	0.0	0.0	0.0	0.0	0.1	0.0	0.0	0.0	0.0	0.0	0.0	0.0	0.0	0.0	0.0	0.0
	Chordata	0.0	0.0	0.0	0.0	0.0	0.0	0.0	0.0	0.0	0.0	0.0	0.0	0.0	0.0	0.0	0.0	0.0
	Streptophyta	0.0	0.0	0.0	0.0	0.0	0.0	0.0	0.0	0.0	0.0	0.0	0.0	0.0	0.0	0.0	0.1	0.0
Unknown	Unknown	1.2	0.5	0.4	0.4	0.0	0.8	0.6	0.0	0.6	0.6	0.6	1.0	0.5	0.9	0.7	0.0	0.7

***gyrB* genes**

		Stream																
Domain	Phylum	CASC	OPAN	MAHF	RIVE	NGAK	OKUR	RANG	HOTE	KUME	MAKA	OTKH	LUCA	PUHI	OTET	OMAR	OAKL	PAKB
Archaea	Euryarchaeota	1.3	2.3	1.3	1.7	3.1	0.9	1.6	2.3	1.5	1.4	4.9	1.4	1.6	1.6	2.4	2.1	2.2
Bacteria	Acidobacteria	2.6	0.0	2.6	0.0	0.0	1.9	3.3	0.0	0.8	2.8	3.3	2.7	1.6	3.1	2.4	0.2	2.2
	Actinobacteria	7.8	2.3	14.3	10.3	0.0	9.3	11.5	0.0	10.6	11.1	8.2	5.4	4.8	4.7	5.9	10.2	6.5
	Bacteroidetes	11.7	4.7	2.6	0.0	3.1	6.5	3.3	9.1	8.7	8.3	3.3	12.2	4.8	4.7	10.6	7.6	6.5
	Chlamydiae	0.0	0.0	0.0	0.0	0.0	0.0	0.0	0.0	1.9	0.0	0.0	0.0	1.6	1.6	0.0	1.2	0.0
	Chlorobi	0.0	0.0	0.0	0.0	0.0	0.0	0.0	0.0	0.4	0.0	0.0	0.0	0.0	0.0	0.0	1.2	0.0
	Chloroflexi	0.0	0.0	0.0	0.0	0.0	0.0	0.0	0.0	0.4	0.0	0.0	0.0	0.0	0.0	0.0	0.7	0.0
	Cyanobacteria	3.9	2.3	0.0	3.4	0.0	2.8	0.0	0.0	3.0	1.4	1.6	5.4	0.0	1.6	4.7	5.1	2.2
	Deinococcus-Thermus	0.0	0.0	0.0	0.0	3.1	0.0	0.0	0.0	0.4	0.0	0.0	0.0	0.0	0.0	0.0	0.0	0.0
	Firmicutes	11.7	14.0	9.1	10.3	15.6	13.0	9.8	6.8	16.2	13.9	13.1	12.2	14.3	12.5	11.8	14.5	10.9
	Fusobacteria	0.0	0.0	0.0	0.0	0.0	0.0	0.0	0.0	0.0	0.0	0.0	0.0	0.0	0.0	0.0	0.2	0.0
	Planctomycetes	1.3	2.3	1.3	1.7	0.0	0.9	1.6	2.3	1.1	1.4	1.6	1.4	1.6	1.6	2.4	0.7	2.2
	Proteobacteria	42.9	48.8	54.5	56.9	56.3	50.9	50.8	59.1	44.5	47.2	47.5	41.9	52.4	50.0	44.7	48.3	47.8
	Spirochaetes	0.0	0.0	0.0	0.0	0.0	0.9	0.0	0.0	1.1	0.0	0.0	1.4	0.0	1.6	1.2	0.9	0.0
	Tenericutes	6.5	9.3	6.5	1.7	9.4	3.7	6.6	6.8	3.8	5.6	6.6	5.4	6.3	6.3	5.9	2.8	10.9
	Thermotogae	1.3	0.0	0.0	0.0	3.1	0.9	0.0	0.0	0.4	1.4	0.0	1.4	1.6	0.0	0.0	0.7	0.0
	Unclassified Bacteria	9.1	14.0	7.8	13.8	6.3	8.3	11.5	13.6	5.3	5.6	9.8	9.5	9.5	10.9	8.2	3.7	8.7
